# Cat-transmitted Sporotrichosis, Rio de Janeiro, Brazil

**DOI:** 10.3201/eid1112.040891

**Published:** 2005-12

**Authors:** Armando Schubach, Tânia Maria Pacheco Schubach, Mônica Bastos de Lima Barros, Bodo Wanke

**Affiliations:** * Evandro Chagas Clinical Research Institute, Rio de Janeiro, Brazil

**Keywords:** Sporothrix schenckii, sporotrichosis, epidemiology, transmission, zoonosis, veterinary, cats, dispatch

## Abstract

Sporotrichosis is an emerging zoonosis in Rio de Janeiro, Brazil. From 1998 to 2003, 497 humans and 1,056 cats with culture-proven sporotrichosis were studied. A total of 421 patients, 67.4% with a history of a scratch or bite, reported contact with cats that had sporotrichosis.

Sporotrichosis is caused by *Sporothrix schenckii*, a dimorphic fungus widely found in nature (*1*). Davies and Troy (*2*) reviewed 48 cases of feline sporotrichosis published over a period of 40 years. Little is known about feline sporotrichosis or the role of cats as a source of infection because reports are scarce. Human sporotrichosis has been related sporadically to scratches or bites by animals (*3*).

Since the 1980s, the role of felines in transmission of the mycosis to humans has gained attention among animal owners, veterinarians, and caretakers (*2*). Epidemics involving a large number of persons or wide geographic areas are rare and have been related to an environmental source of infection (*4**,**5*). No epizootics have been reported.

From 1987 to 1997, before the current emergence of sporotrichosis in Brazil, only 13 cases of human sporotrichosis had been recorded at the Evandro Chagas Clinical Research Institute (IPEC) in Rio de Janeiro (*6*). In 1998, the first year of the current outbreak, 9 patients with human sporotrichosis were observed, 3 of whom reported scratches by cats with cutaneous lesions (*7*). Since then, cats with clinically suspected sporotrichosis or human cases of this disease have been studied systematically.

## The Study

The study protocol was reviewed and approved by the research ethics committee and the institutional review board of the Center for Biological Evaluation and Care of Research Animals of the Oswaldo Cruz Foundation. The patient inclusion criterion for humans and cats in this study was isolation of *S. schenckii* in culture. All human patients were treated at the outpatient clinic of IPEC, and the animals were seen at the veterinary outpatient clinic of IPEC.

From 1998 to 2001, 178 human (*8*) and 347 feline (*9*) cases of sporotrichosis were reported to IPEC. Additionally, 101 apparently healthy cats that lived with other cats with sporotrichosis were identified and followed up for 1 year. All data were collected by review of medical charts and recorded on a standardized form.

Most human cases treated at IPEC came from outlying neighborhoods of greater metropolitan Rio de Janeiro, an area with low socioeconomic conditions. Of 178 patients, 156 reported home or professional contact with cats with sporotrichosis, and 97 reported a history of cat scratch or bite. The patients had an age range of 5 to 89 years (median 39). One hundred twenty-two (68%) were women. Housewives (30%) and students (18%) were the 2 most frequently affected groups; 5% of patients were veterinarians.

Fifty-two (28.6%) of the 170 patients showed a positive result on a leishmanin skin test. Of these patients, 38 came from areas with active transmission of American tegumentary leishmaniasis (ATL) (*10*).

We evaluated 148 cats with sporotrichosis for the presence of *S. schenckii*. The fungus was isolated from all cutaneous lesions, 47% (n = 71) of nasal cavity swabs, 33% (n = 79) of oral cavity swabs, and 15% (n = 38) of nail fragment pools (*11*). *S. schenckii* was isolated from the oral and or nasal cavities of 10 of 101 apparently healthy cats that lived with other cats with sporotrichosis.

Coinfection with feline immunodeficiency virus (FIV) or feline leukemia virus (FeLV) was demonstrated in 21.8% of 142 tested cats with sporotrichosis. Antibodies against FIV were detected in 28 cats, FeLV antigen in 2 cats, and both FIV and FeLV in 1 cat (*9*).

A broad spectrum of clinical signs and symptoms was observed in 347 cats with sporotrichosis, ranging from subclinical infection and a single cutaneous lesion with spontaneous regression to fatal systemic forms. The cutaneous-lymphatic form was observed in only 19.3% of the cats, while mucosal involvement of the upper respiratory and digestive tracts was observed in 34.9% and multiple cutaneous lesions in 39.5% (*9*).

**Figure 1:**
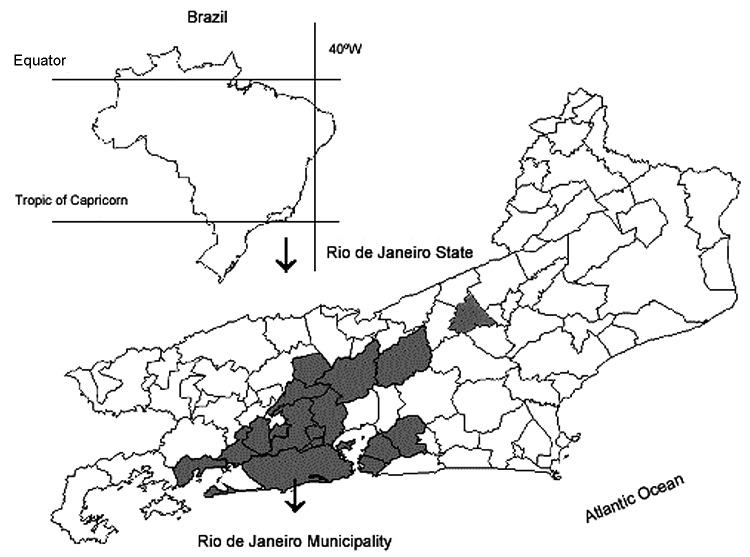
Map of Brazil and the state of Rio de Janeiro showing municipalities (shaded areas) where human and feline cases of sporotrichosis were diagnosed from 1998 to 2003.

**Figure 2:**
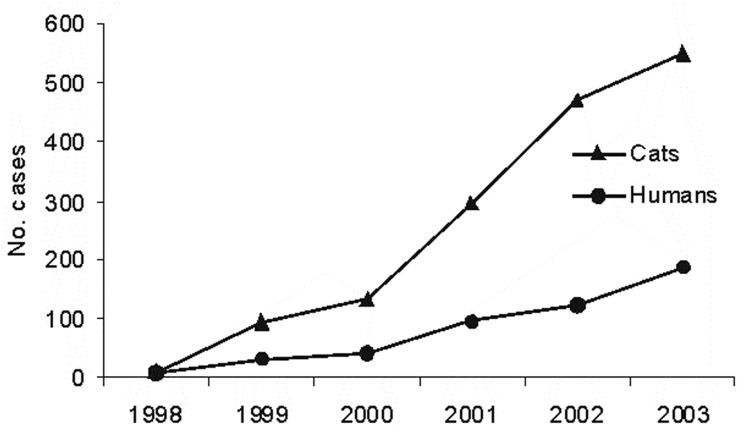
Number of human and feline cases of sporotrichosis diagnosed at the Instituto de Pesquisa Clínica Evandro Chagas, Rio de Janeiro, Brazil, 1998–2003.

We reviewed published data on an ongoing epidemic of zoonotic sporotrichosis in Rio de Janeiro, Brazil. In the first year of this outbreak, 9 cases of human disease and 1 case of animal disease were diagnosed at IPEC. The incidence of sporotrichosis increased so much that by December 2003 a total of 497 humans and 1,056 cats with culture-proven sporotrichosis had been recorded (IPEC, unpub. data) (Figures 1 and 2). A total of 421 patients reported contact with cats that had sporotrichosis; 284 of these patients had a history of a scratch or bite. This finding represents the largest epidemic of this mycosis as a zoonosis. Isolation of the fungus from the nails and oral cavity of cats suggests that transmission can occur through a scratch or bite. In addition, infection may be transmitted through secretions because fungus was isolated from nasal fossae and cutaneous lesions and yeastlike elements were visualized in histologic sections of cutaneous biopsy specimens (*3**,**9**,**12*). The large proportion of housewives among the human patients suggests that this group is the most heavily exposed to the fungus because they care for cats. Molecular typing of *S. schenckii* strains isolated from humans and animals reinforces this hypothesis (*13*).

## Conclusions

The primary differential diagnosis for sporotrichosis was cutaneous leishmaniasis, especially in cases from areas endemic for ATL. In these cases, a diagnosis based only on clinical findings and positive leishmanin skin test result could lead to incorrect treatment and unnecessary control measures (10). In addition to cutaneous infection as a transmission route, the current epidemic also appears to have a strong respiratory component because the frequency of respiratory signs and pulmonary and nasal mucosal lesions was high and because *S. schenckii* was isolated from nasal swabs collected in vivo and from the lungs of autopsied cats (*9**,**11**,**12*).

Some investigators believe that the severity of feline sporotrichosis is related to immunosuppression caused by coinfection with FIV or FeLV (*2*). However, no association with FIV/FeLV-related immunodeficiency was observed (*12*).

The present series consisted mainly of cats with chronic cutaneous lesions whose owners sought specialized care at a reference center. In transmission areas, many cases of subclinical infection and spontaneous cure may have gone undetected. Since reporting sporotrichosis cases is not mandatory, assessing its occurrence and distribution is difficult, and the incidence may have been underestimated. The absence of a feline sporotrichosis control program and various feline behavior factors (e.g., frequent cat fights in the neighborhoods) may have contributed to the spread of the mycosis.

For public health purposes and to control the current epidemic, an effective and viable therapeutic regimen for cats is necessary. In addition, public awareness programs on sporotrichosis prophylaxis are required. These will encourage responsible ownership, neutering, cremation of dead cats, confinement of cats inside the home, limiting the number of cats per household, regular cleaning of dwellings, proper health care for the animals, and general public health measures such as basic sanitation, regular garbage collection, and cleaning of empty lots.
